# Single Cell Sequencing of Induced Pluripotent Stem Cell Derived Retinal Ganglion Cells (iPSC-RGC) Reveals Distinct Molecular Signatures and RGC Subtypes

**DOI:** 10.3390/genes12122015

**Published:** 2021-12-18

**Authors:** Harini V. Gudiseva, Vrathasha Vrathasha, Jie He, Devesh Bungatavula, Joan M. O’Brien, Venkata R. M. Chavali

**Affiliations:** Department of Ophthalmology, Scheie Eye Institute, University of Pennsylvania, Philadelphia, PA 19104, USA; gudiseva@pennmedicine.upenn.edu (H.V.G.); Vrathasha.Vrathasha@Pennmedicine.upenn.edu (V.V.); jhe2@pennmedicine.upenn.edu (J.H.); devesh.bungatavula@gmail.com (D.B.);

**Keywords:** retinal ganglion cells, transcriptome, single cell sequencing, iPSC-RGCs, iPSCs, RGC subtypes, FACS analysis, marker genes, clustering, glaucoma

## Abstract

We intend to identify marker genes with differential gene expression (DEG) and RGC subtypes in cultures of human-induced pluripotent stem cell (iPSC)-derived retinal ganglion cells. Single-cell sequencing was performed on mature and functional iPSC-RGCs at day 40 using Chromium Single Cell 3’ V3 protocols (10X Genomics). Sequencing libraries were run on Illumina Novaseq to generate 150 PE reads. Demultiplexed FASTQ files were mapped to the hg38 reference genome using the STAR package, and cluster analyses were performed using a cell ranger and BBrowser2 software. QC analysis was performed by removing the reads corresponding to ribosomal and mitochondrial genes, as well as cells that had less than 1X mean absolute deviation (MAD), resulting in 4705 cells that were used for further analyses. Cells were separated into clusters based on the gene expression normalization via PCA and TSNE analyses using the Seurat tool and/or Louvain clustering when using BBrowser2 software. DEG analysis identified subsets of RGCs with markers like *MAP2*, *RBPMS*, *TUJ1*, *BRN3A*, *SOX4*, *TUBB3*, *SNCG*, *PAX6* and *NRN1* in iPSC-RGCs. Differential expression analysis between separate clusters identified significant DEG transcripts associated with cell cycle, neuron regulatory networks, protein kinases, calcium signaling, growth factor hormones, and homeobox transcription factors. Further cluster refinement identified RGC diversity and subtype specification within iPSC-RGCs. DEGs can be used as biomarkers for RGC subtype classification, which will allow screening model systems that represent a spectrum of diseases with RGC pathology.

## 1. Introduction

Glaucoma is a group of eye diseases that damages the optic nerve and is the leading cause of irreversible blindness worldwide [1]. Glaucoma is caused by multiple risk factors—such as high intraocular pressure (IOP), high blood pressure, diabetes, and genetic predisposition [2]. During advanced glaucoma, blurred vision is induced by the degeneration of retinal neurons and the death of retinal ganglion cells (RGCs). RGCs are diverse and differ in their “physiological roles exhibiting varied responses to visual stimuli,” containing multiple subtypes [3]. Since the mechanisms leading to the RGC degeneration are unclear, there are no known treatments to reverse this irreversible vision loss. Supplementary treatments are quite limited and range from pharmacological to surgical mitigation of intraocular pressure, such as eye drops, laser surgery, trabeculoplasty, etc. [4]. Due to this complex and temporary rescue, researchers are looking for other pathways that may provide promising and permanent treatments. Scientists have recently explored using induced pluripotent stem cells for modeling glaucoma in a dish and for studying glaucomatous degeneration [4]. Stem cells have self-regeneration ability and improved differentiation methods to generate highly purified and functional induced pluripotent stem cell derived RGCs (iPSC-RGCs) are essential for studying RGC cell diversity [3,5]. However extensive classification of human RGC subtypes has not been performed due to limited availability of human retinal tissues and very few studies on characterizing molecular signatures in iPSC-RGCs.

The use of single-cell sequencing technology can help with identifying smaller distinct cell populations and outline cell maps. The advantage of single-cell transcriptomics compared to conventional bulk-RNA Seq methods is that unique markers can be found in many unknown cell subtypes [6]. RNA-sequencing methods include the isolation of a single cell (scRNA-seq) from a group of cells, followed by RNA extraction and amplification, and later processing to study cell mapping, cell segregation and cell classification [7]. In addition to scRNA-seq analysis, clustering approaches are crucial in classifying cells based on their differential gene expression profiles. More recently, various clustering approaches, such as hierarchical clustering, K-means clustering, SNN-Cliq, pcaReduce, SC3, Seurat, SCANPY, RCA, and dropClust, were used to separate single cells based on differential gene expression and similarity [8].

Methods have been published over the past few years trying to modify RNA-seq approaches to capture more information and identify molecular biomarkers. Some studies have focused on performing single-cell RNA-Seq in the vertebrate retina to understand cell populations of the central nervous system. One study analyzed murine bipolar cell markers from microarray hybridization to RNA-Seq to study their evolution, providing “a more in-depth examination of larger numbers of cells at a decreased cost” [9]. In addition, other researchers have utilized other techniques such as FAC-Sorting, imaging analyses, and electrophysiology for improved cell examination [9]. By finding expressed genes in populations of Parvalbumin-expressing cells, researchers detected possible candidates for ipRGC classification such as *Prph*, *Ctxn3*, and *Prkcq* in RGCs derived from embryonic stem cells [9]. A recent study also summarized the complexity of retina and pluripotent stem cell derived retinal organoids with single cell RNA sequencing [10]. This methodology uses a combination of single-cell profiling, hierarchical clustering, and statistical analysis. Although these techniques have made great strides in determining numerous RGC subtypes, many challenges remain in the preparation of cells leading up to sequencing. One standardized method of RGC isolation involves isolating cells from Retinal Organoids using manual dissociation methods and using hierarchical clustering for cell classification. Such methods may introduce loss of mRNA, increase stress among cells and produces tissue damage during cell preparation and cause disruption of neuronal activity [6]. Validation of such data to find correlation of a genetic marker to a functional phenotype is often challenging [6].

To further understand the subtypes of RGCs and determine if we can differentiate iPSCs into RGC subtypes, we performed single-cell sequencing on purified and functional iPSC-RGCs obtained from a standardized two-step RGC differentiation protocol [5]. To address shortcomings from previous methods, our protocol takes a holistic approach to identify marker genes, differential gene expression and retinal ganglion cell (RGC) subtypes in human iPSC-RGCs with minimal disruption. Specifically, by using 10X Genomics, single-cell RNA sequencing and Chromium Single Cell 3′ library preparation, we analyzed transcriptomes on a cell-to-cell base level. This then helped in identifying unique, modern-based markers to discern RGC subtypes within our iPSC-RGC clusters. Identifying the genes that code for these RGC specific subtypes will allow for better comprehension of the diversity within the retina [3]. Further characterization of iPSC-RGC subtypes will deepen our understanding of how these cells differ and how they are “affected in disease states” and under different experimental conditions.

## 2. Materials and Methods

*Differentiation of iPSC-RGC cultures:* Our lab has a repository of human iPSCs that were generated from keratinocytes or blood cells via polycistronic lentiviral transduction (Human STEMCCA Cre-Excisable constitutive polycistronic (OKS/L-Myc) Lentivirus Reprogramming Kit, Millipore) and characterized with a hES/iPS cell pluripotency RT-PCR kit [11]. The RGCs for our studies were derived using small molecules to inhibit BMP, TGF-β (SMAD) and Wnt signaling to differentiate retinal ganglion cells (RGCs) from iPSCs. The iPSCs were differentiated into pure iPSC-RGCs cells with structural and functional features characteristic of native RGC cells as described previously in Chavali et al., 2020, by Day 36 of differentiation [5]. iPSC-RGCs were further matured and used for single-cell sequencing studies at Day 40.

*FACS analysis:* iPSC-RPCs and iPSC-RGCs cultures were lifted using TrypLE Express (Invitrogen, catalog #: 12605-010, Waltham, MA, USA) and collected by centrifugation at 1600 rpm for 5 min at 4 °C. The pelleted cells were resuspended in 1X PBS supplemented with 0.5% bovine serum albumin and 0.1% sodium azide (FACS buffer). Cells were fixed in 4% paraformaldehyde (PFA) (*v*/*v*) for 15 min at room temperature (RT) followed by permeabilization using 0.5% Tween-20 (*v*/*v*) for 10 min at RT. Cells were incubated with various antibodies: anti- Ki67/MK167 (Novus Biologicals, catalog #: NB500-170SS, Centennial, CO, USA), anti-Chx10 (Millipore, catalog #: AB9016, Burlington, MA, USA), anti-CD90 (Thy1; Novus Biologicals, catalog #: AF2067, Centennial, CO, USA), anti-sheep Alexa Fluor 405 (abcam, catalog #: ab175676, Cambridge, MA, USA), anti-BRN3 Alexa Fluor 594 (Santa Cruz, catalog #: sc-390780, Santa Cruz, CA, USA), anti-SNCG Alexa Fluor 488 (Santa Cruz, catalog #: sc-65979, Santa Cruz, CA, USA), and anti-RBPMS Alexa Fluor 647 (Novus Biologicals, catalog #: NBP273835AF647, Centennial, CO, USA). Stained cells were analyzed using LSR B and LSRFortessa B at Penn Cytomics and Cell Sorting Resource Laboratory. Data were further analyzed using the FCS Express software.

*Single Cell Preparation of iPSC-RGCs:* The iPSC-RGCs at Day 40 grown on 10 cm Matrigel coated plates were dissociated by incubation with 5 mL of Accutase (Millipore Sigma, Cat #A6964, Burlington, MA, USA) for 10 min. The cell suspension was centrifuged at 300× *g* for 5 min and the cell pellet was washed with 1X HBSS before suspending them in 1X PBS with 0.04% BSA. Single cell suspension was prepared by mixing the cells gently using a wide-bore glass tip and the cell count was determined using Countess II automatic cell counter (Life Technologies, Carlsbad, CA, USA). Single cells in suspension were also analyzed for their viability using a hemocytometer and Trypan Blue staining.

*Generation of single cell gel beads in emulsion (GEM) and Sequencing libraries:* Single cell suspensions of mature and functional iPSC-RGCs at day 40 were diluted to required concentrations and loaded onto 10X Genomics Single Cell 3′ Chips as per Chromium Single Cell 3’ V3 kit following manufacturers protocol. Single cells were partitioned using chromium controller (10X Genomics) into GEMs containing unique barcoded primers with unique molecular identifier (UMI), followed by cell lysis, reverse transcriptase (RT), amplification of barcoded cDNA and fragmentation to nearly 200 bp and sample indexing. cDNA libraries were quantified using the KAPA library Quantitation kits following manufacturer’s instructions (KAPA Biosystems, Wilmington, MA, USA).

*Single Cell Data Sequencing and Data Analysis:* Sequencing libraries were prepared from 8008 cells and were run on Illumina Novaseq 6000 in two lanes to generate 150 PE reads. The CellRanger 4.0.0 pipeline (10X Genomics) analysis was used to demultiplex raw base calls from FASTQ files, performs alignment, filtering, barcode and unique molecular identifier (UMI) counting. The demultiplex FASTQ files were mapped to the hg38 ref genome using STAR package. The output from 10X Genomics Cellranger 4.0.0 pipeline was used as input into the R analysis package Seurat version 3.2.2. Cells with high unique molecular index counts (nUMI), high mitochondrial transcript load were filtered out from the analysis. The data were then normalized, scaled, and explored using Seurat’s recommended workflow. Principal component analysis (PCA), Louvain clustering, and the Uniform Manifold Approximation and Projection (UMAP) were performed. Normalized gene expression was obtained by LogNormalize in Seurat using a scale factor of 10,000 in which feature counts for each cell were divided by the total counts for that cell and multiplied by the scale factor. The value was then transformed to natural-log using log1p. We performed cluster-to-cluster differential expression of the formed clusters and tested using the Wilcoxon-Rank Sum to identify unique gene markers for each cluster. QC analysis was performed after aggregating datasets in both lanes by removing the reads corresponding to ribosomal (>50%) and mitochondrial genes (>20%), as well as cells that had a mean absolute deviation (MAD) lower than 1X. Sub-populations of single cell clusters were identified using unsupervised clustering. Differential expression gene (DEG) analysis of the identified clusters was performed to characterize subsets of RGCs and their markers in the iPSC-RGCs. Gene expression patterns for marker genes among clusters was also performed.

## 3. Results

### 3.1. Characterization of iPSC-RGCs with FACS Analysis to Determine RGC Purity

**Figure 1:**
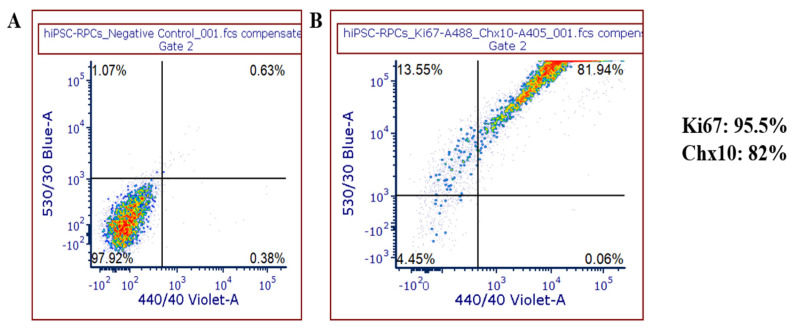

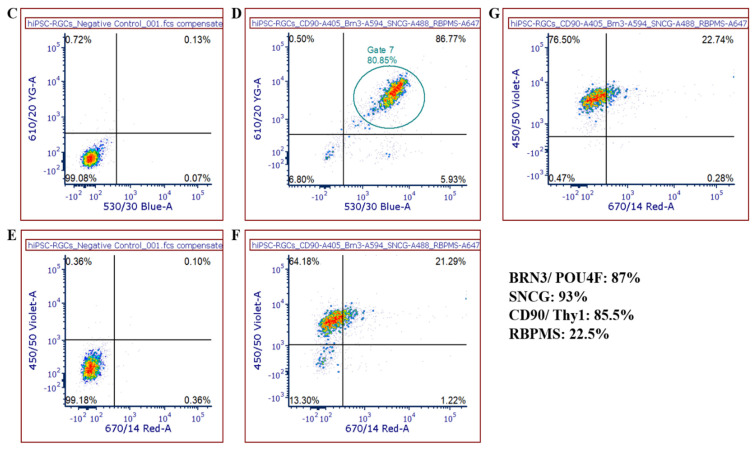
Flow cytometry analysis to characterize iPSC-RPCs and iPSC-RGCs. (**A**,**B**) FACS analysis of iP SC-RPCs at day 15 showed that over 95.5% and 82% of the cells expressed ki67 and Chx10, respectively. About 82% of the cells expressed both the RPC markers. (**C**–**F**) FACs analysis at day 35 showed that 87%, 93%, 85.5%, and 22.5% of the iPSC-RGCs were positive for BRN3, SNCG, CD90, and RBPMS, respectively. (**G**) A subset of the iPSC-RGC population (insert from (**D**)) expressed all the four RGC markers; namely 87% of the cells were positive for BRN3 and SNCG, whereas 81% was positive for CD90 and 19% was positive for RBPMS.

We differentiated human iPSCs, using a standardized two-step methodology involving inhibition of SMAD and Wnt pathways [5]. During differentiation, the retinal progenitor cells (RPCs) were characterized for RPC expression markers by Day 15 and for RGC expression markers by Day 35 using FACS. Our protocol generated nearly 100% RPCs with majority of them staining positive for Ki67 (over 95%) and Chx10 (82%) (Figure 1A,B) by FACS sorting. Other studies reported to date mainly used Thy1 cell surface marker for FACS sorting; a subset of the RGCs that are positive for CD90. In addition to CD90, we characterized RGCs with established RGC markers. FACS analysis showed positive staining for Brn3 (87%), SNCG (93%), RBPMS (22.5%) and CD90/Thy1 (85.5%) antibodies among the total cells differentiated from iPSCs (Figure 1C–F). Further analysis of a subset of the iPSC-RGC population expressing all the four RGC markers, showed that 87% of the cells were positive for BRN3 and SNCG, whereas 81% was positive for CD90 and 19% was positive for RBPMS (Figure 1G). Well differentiated iPSC-RGCs are characterized by FACS, were further verified by ICC using RGC specific antibodies and quantitative reverse transcription PCR profiles for RGC expressing gene transcripts as described previously [5]. The qRT-PCR profiles identified different transcripts expressed in iPSC-RGCs indicating the diversity of the gene expression in iPSC-RGCs.

### 3.2. iPSC Differentiation Generates Several RGC Subtypes

Differentiated iPSC-RGCs show extensive morphological features including healthy cell bodies and elongated axons forming bundles as early as day 32 of differentiation and cells form elongated clusters in vitro on Matrigel coated plates as they mature [5]. Treatment with Accutase for dissociation into single cells did not affect their viability when replated on Matrigel coated plates. To characterize RGC transcriptional profiles and distinct RGC subtypes, we performed single cell sequencing (10X Genomics sequencing platform) on iPSC-RGCs at Day 40. Our isolation procedures generated single cell iPSC-RGCs suspension of over 9000 cells for sequencing on an Illumina platform with over 95% viability. Processing of data for single cell sequencing resulted in sequences generated from 8008 cells with over 22,000 genes detected per cell (Table S1). Cells with mitochondrial reads above 20% and with ribosomal reads above 50% were removed from our data analysis pipeline. A total of 4556 cells with 1X MAD that passed quality controls were analyzed for study from Day 40.

**Figure 2:**
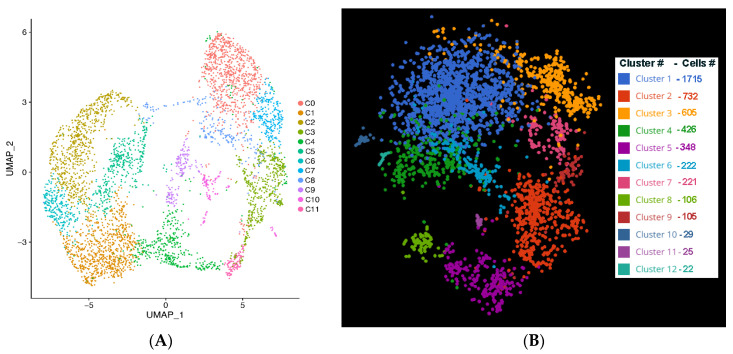
scRNA-Seq analysis of iPSC-RGCs at differentiation Day 40. Uniform manifold approximation and projection (UMAP) clustering resulted in 11 overlapping clusters of iPSC-RGCs (**A**). Graph based clustering of 4556 cells after initial QC and filtering shows cells organized into 12 clusters as shown in this representation using BBrowser2 software (**B**). The number of cells in each cluster is represented in Figure 2B against each cluster.

The medium sequence reads were 28,667 per cell. The medium number of genes captured were 22,866 with >97% of reads mapped to the genome. UMAP clustering was performed with the Seurat 3.1.1. Marker gene analysis was used to identify the clusters. The cells that passed QC were segregated into 12 clusters based on gene expression normalization via PCA and TSNE analysis in Seurat. We also used Bioturing BB browser2 to visualize the clusters and find marker genes and DE analysis downstream [12]. Nine clusters totaling to 98% of the cells were used by BB browser, three clusters with less than 1% of the cells were not included. The largest cluster, Cluster 1 consists of 1715 cells and the smaller cluster in our analysis consists of 105 cells. The clusters are color coded for easy recognition (Figure 2B). 

**Figure 3:**
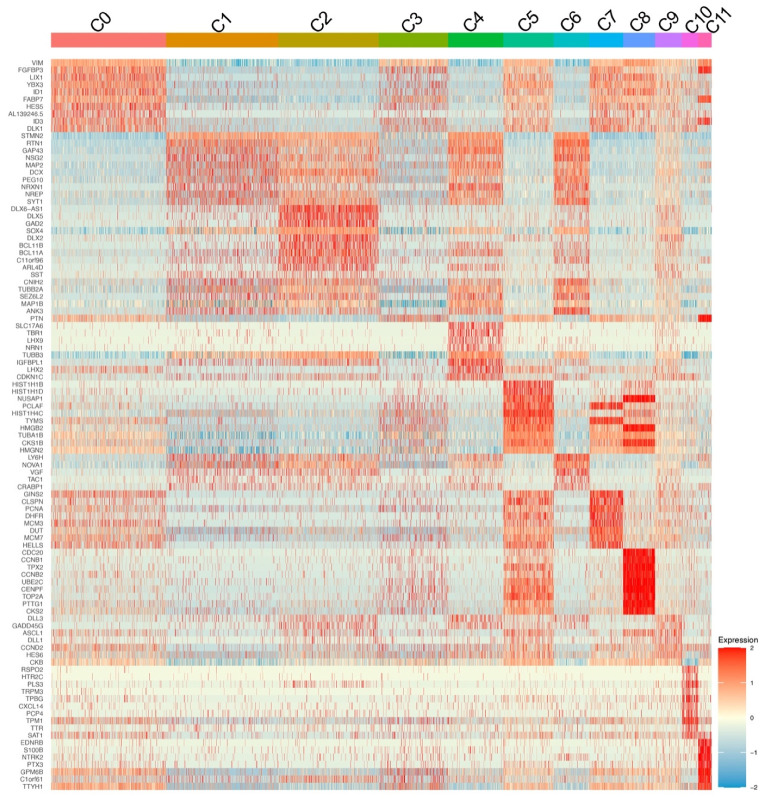
Heatmap showing top ten enriched genes in each cluster. Each horizontal line represents one gene, and each vertical line represents one cell. The heatmap demonstrates continuity between these clusters. The relative expression levels that were generated by averaging the normalized gene expression values in each cluster are shown in color scale.

Heatmap showing 10 marker genes for each cluster, highlighted the gene clusters that are highly expressed and segregated based on gene expression profiles among the nine clusters. BB browser1 analysis was used to identify cluster specific marker genes. A set of genes with higher expression in RGCs like *TUBB3*, *BASP1*, *SOX4*, *STMN2, TUBA1A*, *TUBB2B*, *EIF4A1*, *VIM*, *MARCKSL1*, *HMGB1* and *CKB* are predominantly expressed among all clusters (Figure 3). Majority of these genes are established RGC markers like *TUBB3*, *STMN2*, and few are known for their role in regulating actin cytoskeleton like *BASP1*.

Apart from the above genes, expression of markers in Cluster 2 (732 cells) and Cluster 5 (348 cells) appear to be similar for *PTN*, *TTYH1*, *GPM6B*, *FGFBP3* and *HMGB2* genes. *PTN* is known to play an important role in cell proliferation, adhesion, and migration of neuronal cells during ocular development [13]. *GPM6B* has a role in stabilizing axonal membrane. Clusters 3 (606 cells) and 7 share a set of three genes with similar expression profiles to those from Cluster 7 (221 cells) in expressing *MLLT11*, *KLF5C* and *GAP43* genes. Cluster 3 also has an extremely high expression of SST gene when compared to the remaining clusters. SST is a neurotransmitter and plays a role in the development of RGCs. SSTs are also expressed in GABAergic neurons. *MLLT11* expression is differentially expressed in maturing neurons especially restricted to TUJ-1 positive cells [14]. GAP-43 gene is implicated in regulation of presynaptic vesicular function, axonal growth, and plasticity [15]. It is known to mediate retinal axon interaction during optic tract formation [16].

In addition to the RGC specific genes, Cluster 8 (106 genes) have enriched expression of *HMMR*, *PTTG1*, *UBE2C*, *CENPF*, *TOP2*, *CSK1B*, *CCNB1*, *CCNB2* and *CDC20* genes. *CCNB1* and *CCNB2* work in opposition to coordinate the self-renewal and lineage commitment of RGCs. Majority of genes in this cluster have roles in cell cycle regulation, cell division and cellular/neuronal reprogramming.

### 3.3. Enrichment of Marker Genes between iPSC-RGC Clusters

To further examine the features of the individual clusters, we looked at the marker gene expression of each cluster and compared with all the other clusters to identify genes that are differentially expressed in individual clusters (Table S2). Differential expression analysis (average log fold change > ±0.5 and *p*-val-adj < 0.05) between the separate clusters identified DEGs.

We examined the expression of the top ten enriched marker genes as organized by *p*-values from each cluster and represented the data by a heatmap (Figure 3). This analysis did not only confirm their enrichment of marker genes in the corresponding clusters but also revealed genes expressing across other neighboring clusters, suggesting the association and continuity among these clusters.

The top 10 enriched genes in Cluster 1 are also upregulated in Clusters 4 and 6, indicating that the clusters are likely linked. Genes like *RTN1* and *PEG10* are overexpressed in Clusters 1 and 6. The function or role of these two genes in RGCs or glaucoma is not established to date. *RTN1* transcript is significantly downregulated in Clusters 0, 5, 7, 8 and 11. *STMN2* is only upregulated in Clusters 4 and 6 but it is found to be the most downregulated gene in Clusters 0, 3, 7, 8, 10 and 11. For Clusters 1, 2, 4 and 6 the most downregulated gene when compared to other clusters was *VIM*, whereas it was the most overexpressed gene in Cluster 0. *GAP43* was found downregulated in Clusters 5, 7 and 10 but it is upregulated in Cluster 2 and 4. *FABP7* plays a role in fatty acid uptake, transport, and metabolism. *FABP7* was found only among the upregulated genes in 0, 3 and 11 clusters and downregulated in 1, 2 and 6 clusters. *IDI1* and *ID3* are negative regulators of TGFβ pathway. *ID1* is overexpressed in Cluster 0 and downregulated in Cluster 4. ID3 is overexpressed in Cluster 0 and downregulated in Clusters 2, 4 and 6. *DLK1* is overexpressed in Cluster 0 and downregulated in Clusters 2, 6 and 11. *MAP2* is overexpressed in Cluster 1 and downregulated in Clusters 5 and 7. *MAP2* is widely used to identify neuronal cells and facilitates in stabilizing microtubules, organelle transport in axons and dendrites. We also observed higher expression of neuronal marker genes like *TUBB3*, *MAP2*, and *TUBA1A* in several clusters (Figure 3).

### 3.4. Distribution of Previously Reported RGC Marker Genes

**Figure 4:**
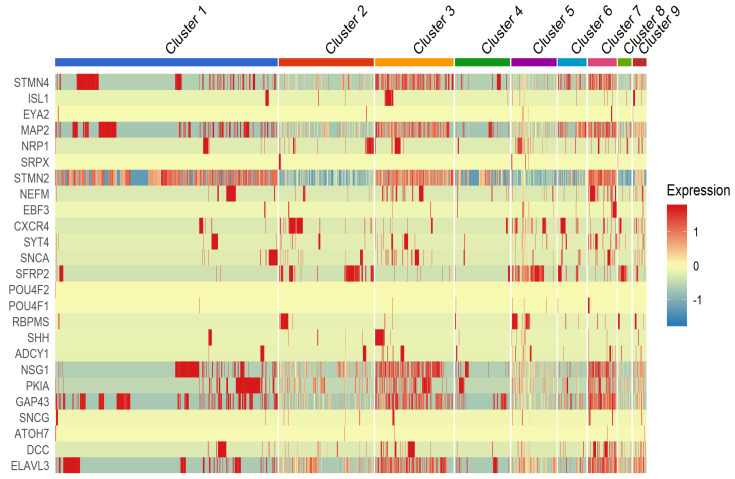
Heatmap showing RGC-specific marker gene expressions transformed in to Z-score in all clusters from single cell sequencing. Corresponding color key histograms are displayed.

In addition to cluster specific marker genes, we interrogated if single cell clusters express genes previously reported in RGC subtype populations [4,9,17]. The RGC specific genes, *STMN2*, *STMN4*, *GAP43*, *MAP2*, *ELAVL3*, *NSG1* and *PK1A* gene transcripts are expressed in majority of the clusters as shown in Figure 4. *DCC*, *SNCA*, *CXCR4*, *STY4*, *SHH*, *ADCY1*, *ISL1*, *RBPMS*, *SNCG* and *EBF3* were expressed in Clusters 2–9 with a lesser intensity. Expression of established neuronal markers in the several clusters indicates that differentiated iPSC-RGCs exhibit genes characteristic of RGCs.

**Figure 5:**
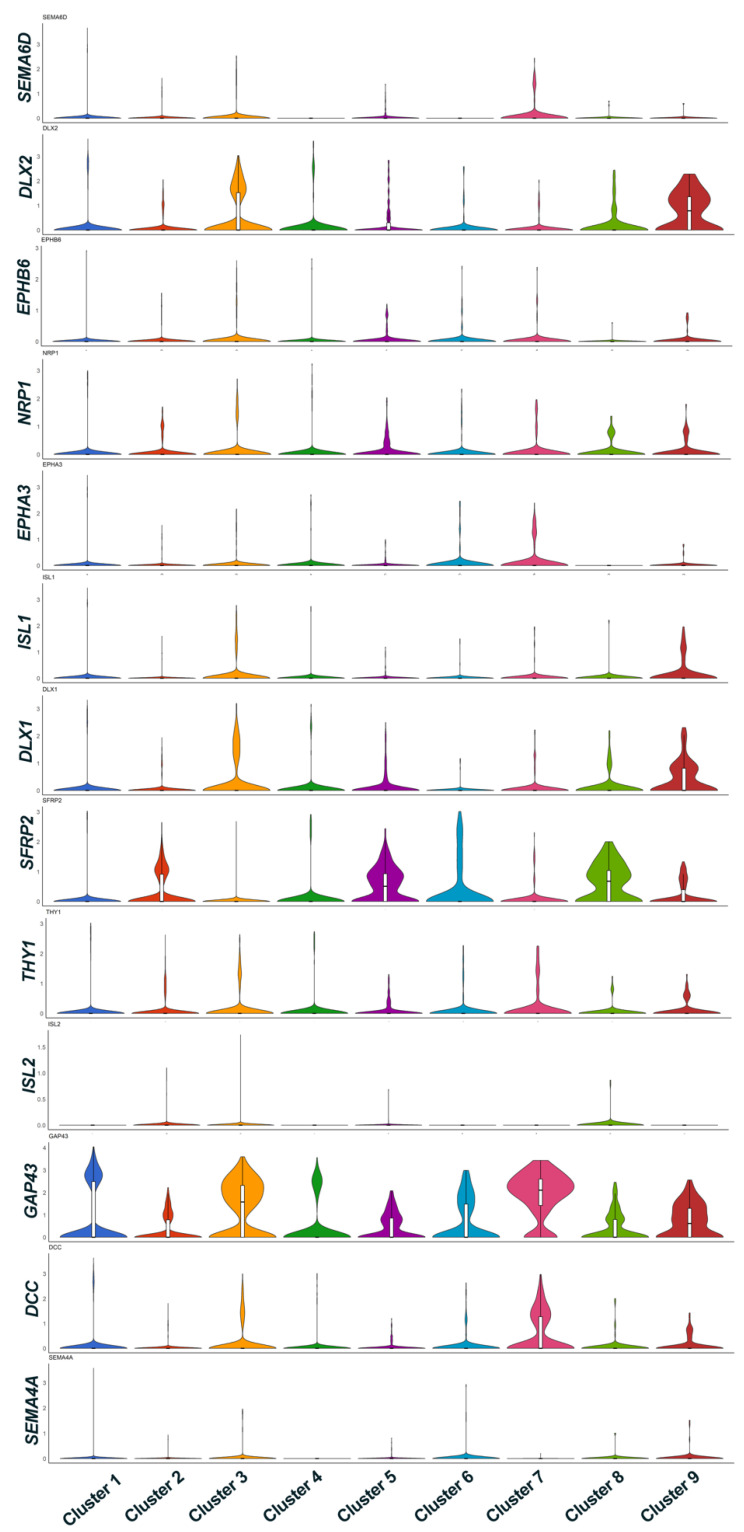
Violin plots showing RGC maturation markers expression.

We compared the RGC clusters for their expression of maturation markers and axon representative genes among the Clusters. The *DLX2*, *EPHB6*, *NRP1*, *EPHA3*, *DLX1*, *GAP43*, *THY1* and *DCC* genes are expressed in majority of clusters with different intensities (upregulation/downregulation) among clusters as seen in violin plots (Figure 5). Clusters 3 and 9 show predominant expression of *DLX1* and *DLX2* which are known to regulate BRN3B expression and determine RGC cell fate [18]. *GAP43* gene which is known to mediate RGC axon interaction is also known to be expressed in these clusters. Upregulation of *SFRP2*, an early marker of RPCs is observed in Clusters 5, 6 and 8. This secreted frizzled related protein is known to modulate pathfinding of mouse RGC axons [19]. *SEMA6D* is upregulated in RGCs in Clusters 3 and 7 along with *GAP43* and *DCC* genes indicating that these clusters express genes supporting mature RGC axon growth, assist in axon guidance, interaction, and survival [20]. The genes in the above clusters are directly relevant to the establishment and maintenance of the RGC identity; and genes encoding regulatory, functional, and structural proteins are critical for RGC differentiation and RGC maturation and axon extension and interaction (Figure 3). These observations are further confirmed by GO analysis.

The single-cell RNA-seq analysis identified different classes of cells and markers among the iPSC-RGCs. A GO analysis of the markers for each cluster indicated the potential nature of each cluster (Figure 2A). For iPSC-RGCs grouped in Cluster 0, GO analysis states that these cells were enriched for regulation of microtubule cytoskeleton organization, intracellular transport, regulation of neuron projection development, axonogenesis and neural nucleus development (Table S3). Cluster 1 has RGCs that participated in the regulation of neurogenesis, cell development and glial cell development. Cluster 3 is specific in that all the cells in this cluster participated in axon development (q value = 8.06 × 10^4^), regeneration, axonogenesis, membrane localization and gliogenesis. Cells in Cluster 4 has iPSC-RGCs that participated in axon development (q value = 9.99 × 10^6^), neuron migration, axon guidance, neurogenesis and regulation of nervous system development (q value= 3.4 × 10^3^). Markers in Clusters 5, 7 and 8 were enriched for cell division process (q value = 1.08 × 10^15^), chromatin remodeling (q value = 7.7 × 10^4^) and markers involved in glial cell development (q value = 8.9 × 10^3^). Few cells in Cluster 8 also showed markers involved in axonal transport (q value= 7.8 × 10^3^), microtubule transport and gliogenesis (5.5 × 10^3^). Cluster 6 contained cells that were enriched for protein targeting to membrane, axonal transport (q-value = 9.8 × 10^6^), transport along microtubules (q-value = 3.12 × 10^4^) and axon development (q value = 1.4 × 10^4^). Markers of Cluster 9 were significantly enriched in regulation of neurogenesis (q value = 3.03 × 10^3^), notch signaling pathway (q value= 4.4 × 10^3^) and neuron fate specification. Cells in Cluster 10 has RGCs that participated in axonogenesis (q value = 5.16 × 10^5^), axon guidance, extracellular matrix organization (q value = 3.2 × 10^3^) and oligodendrocyte differentiation. Cluster 11 has iPSC-RGCs that were involved in neuron differentiation (q value = 4.13 × 10^4^), neuron migration and axon development (q-value = 1.9 × 10^3^) (Table S3).

**Figure 6:**
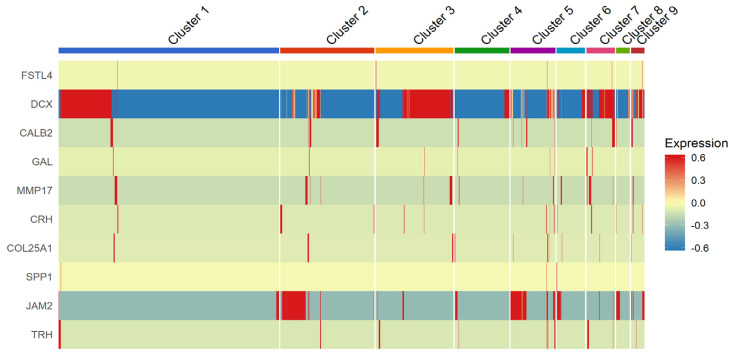
Heatmap showing RGC-subtype specific expression in iPSC-RGC clusters. The expression is transformed to z scores and represented. Corresponding color key histograms are displayed.

Along with known marker genes reported in previous RGC transcriptome studies, we also identified several RGC subtypes^2,3^ based on gene expression. We determined the gene expression profiles of 10 maker genes corresponding to RGC subtypes like *DCX* (ON-OFF DSRGCs), *JAM2* (J-RGCs), *CALB2* (OFF RGCs), *MMP17*, *TRH*, *CRH*, *COL25A1* (ON-OFF RGCs), *GAL* (OFF RGCs), *FSTL4* (ON DSRGCs) and *SPP1* (OFF RGCs) [4]. Among the RGC subtypes, DCX, which is a marker for ON-OFF direction select RGCs is predominantly expressed in iPSC-RGCs segregated in Clusters 1, 3, 4, 7 and 9. Additionally, *JAM2,* which is a marker for J-RGCs (OFF-RGCs) is also expressed in Clusters 2 and 5. Among other RGC subtypes, iPSC-RGCs expressing *FSTL4* and *SPP1* (ON-RGCs) is seen in least numbers indicating that iPSC-RGC differentiation produces least number of ON-subtype RGCs (Figure 6).

## 4. Discussion

The iPSC derived RGCs will serve as unique cellular model systems to study the pathobiology of RGCs in conditions of ganglion cell loss like optic neuritis and primary open angle glaucoma. Our unique iPSC-RGC differentiation strategy involves two stages where iPSCs are initially differentiated to RPCs and then to RGCs [5]. The RPCs generated in our protocol showed highest amounts of expression for Ki67+ and Chx10+ markers by flow cytometry, which is a characteristic of proliferative cells. Expression of these markers in addition to *RAX, CRX* and *PAX6* marker expression confirms the photoreceptor precursor identity of these cells [5]. iPSC-RGCs generated from our protocol form extensive neurite projections and axonal bundles with cell bodies as early as Day 2 after crosshatching (Day 26) in the maturation media. Less than 5% of the differentiated neuroretinal progenitor cells appear to regain differentiation through mitosis in the late stages of iPSC-RGC maturation (after Day 40). Preliminary studies in our lab have shown that using anti-mitotic inhibitors like AraC prevented the growth and proliferation of these mitotic cells, thus promoting iPSC-RGC purity and maturation [21]. Inhibition of SMAD and Wnt pathways generated enriched iPSC-RGC populations showing electrophysiological response like functional RGCs by Day 35.

The differentiated iPSC-RGCs consist of several RGC subtypes which can be identified based on their gene expression profiles, electrophysiology and morphology. Recent studies also showed differences in the susceptibility and resistance of iPSC-RGC subtypes to insults [17,22]. iPSC-RGCs exhibit unique gene expression markers that distinguishes them from other retinal subtypes and serve as molecular biomarkers. We demonstrate that iPSC-RGCs can be identified by expression of high amounts of *BRN3B*, *THY1*, and *SNCG* markers with over 85% of iPSC-RGCs staining for all three markers. Like other reported studies, we found that our iPSC-RGC populations express low levels of RBPMS [3]. Hence, staining with RBPMS is not recommended for assessing percentages of mature RGCs from differentiation. Although RBPMS is considered a pan-RGC marker, its role in RGCs suggests that it may be an excellent marker for RGCs during degeneration [23]. Increased expression of RGC markers shows that our differentiation methodology yields high percentage of mature iPSC-RGCs.

The clustering software used in our study (either with UMAP using Seurat or Louvain clustering in BBrowser2 software) segregated iPSC-RGCs into 11 clusters based on DEGs [12]. Clusters with higher expression of RGC marker genes are predominant among all clusters indicating that differentiated iPSC-RGCs cells belong to RGC lineage. SRCCA analysis showed that majority of the iPSC-RGC clusters reported high expression of RGC specific genes like *MAP2*, *GAP43*, *STMN2*, and *STMN3*. Genes expressed in other clusters play a role in cell adhesion, neuronal cell migration, neuronal maturation, RGC lineage commitment and retinal axonal interaction. Expression of RGC characteristic genes like *RBPMS*, *SNCG* and *ISL1* are expressed with a lesser intensity indicating that varied transcript expression in iPSC-RGCs may be characteristic of RGC subtypes. Violin plot representation of maturation markers and axon representative genes showed cluster specific differential expression of genes for genes related to RGC axon interaction (*GAP43*), axon guidance and survival (*SEMA6D* and *DCC* genes). Genes that are known to regulate Brn3B expression are also differentially expressed in selective clusters (*DLX1* and *DLX2*). Clusters with DEG (up/down-regulation) related to maturation markers and axon extension reveal the diversity of RGC subtypes. The RGC subtype classification in humans is poorly understood. A recent study on human iPSC-RGCs using single-cell qRT-PCR for known RGC- and RGC-subtype-specific markers, reported expression of transcripts corresponding to DS-RGCs, α-RGCs, and IP-RGCs [3]. In our study, we determined that majority of the iPSC-RGC belonged to ON–OFF direction select RGCs along with few RGC subtypes showing greater heterogeneity among RGC clusters. We also observed the presence of ON- and OFF-RGC subtypes segregated into few clusters indicating that differentiated clusters comprised of more than one RGC subtype.

GO analysis of markers genes for individual clusters showed that iPSC-RGC cells in these clusters are enriched for processes responsible for neuronal transport, axonogenesis and neuronal function. Only a few cells in Clusters 5, 7 and 8 showed marker genes for that enriched for cell division, chromatin remodeling and glial cell development. These clusters may have some cells RPCs or immature cells with neuronal origin as evidenced by the expression of SFRP2 in these clusters.

Strengths of our study include using highly purified unmodified iPSC-RGCs with little processing from a 2D culture with high viability before single cell sorting and RNA sequencing. A limitation of our study is that we classified iPSC-RGCs subtypes only based on expression of subtype specific molecular markers and their segregation in clusters based on gene expression. However, further characterization of RGC subsets using electrophysiology (functional) and dendritic branching (phenotypic) is warranted to identify RGC subtypes. However, future studies in our lab are aimed at further characterizing RGC subtypes based on DEG expression using qRT-PCR and immunohistochemistry. Additionally, transcriptome data from day 40 will be compared with single sequencing data obtained from iPSC-RGCs matured until days 74 and 110. Future studies must analyze the susceptibility of iPSC-RGC subtypes to oxidative stress, senescence, and excitotoxicity. DEGs associated with these induced conditions will identify RGC subtype specific expression/response/resistance to these induced conditions. The well-characterized iPSC-RGCs with a specific subtype susceptible for excitotoxicity and oxidative stress can be used as a promising cell based therapeutic approach to treat glaucoma by replacing degenerating RGCs with iPSC-RGCs in models of induced ganglion cell loss. Our studies will unravel pathways and mechanisms leading to ganglion cell loss and inform novel strategies for neuroprotection for all RGC subtypes.

## 5. Conclusions

Several subtypes of iPSC-RGCs are identified based on their transcriptome profiles. Interestingly the transcriptomes of all iPSC-RGCs were obtained from normal differentiated iPSCs without gene manipulation and may vary with the development or maturation of individual cells. Single cell sequencing analysis identified both known and novel marker genes to distinguish subsets of RGCs in our iPSC-RGC clusters. The differentially expressed genes can be used as biomarkers for RGC subtype classification within the human retina. Understanding the RGC diversity will aid in the identification of RGC subsets that may be susceptible to early degeneration during different stages of glaucoma and optic neuropathies. Identification of RGC subtypes that are resistant/resilient and/or susceptible to optic nerve crush and traumatic brain injury may help to tailor precision therapies using neuroprotective drugs/gene overexpression to selective iPSC-RGCs based on molecular markers.

## 6. Patents

The iPSC-RGCs generated in our protocol and improved sorting efficiency of iPSC-RGCs are covered under Penn Center for Innovation Disclosure#22-9778.

## Data Availability

The data presented in this study are available on request from the corresponding author.

## Supplementary Materials

The following are available online at https://www.mdpi.com/article/10.3390/genes12122015/s1, Table S1: RNA sequencing metrics of iPSC-RGCs; Table S2: Top 10 upregulated and downregulated genes from each cluster; Table S3: Results of GO analysis of each iPSC-RGC cluster.
